# Not in wilderness: African vulture strongholds remain in areas with high human density

**DOI:** 10.1371/journal.pone.0190594

**Published:** 2018-01-31

**Authors:** Mohamed Henriques, José Pedro Granadeiro, Hamilton Monteiro, Ana Nuno, Miguel Lecoq, Paulo Cardoso, Aissa Regalla, Paulo Catry

**Affiliations:** 1 MARE–Marine and Environmental Sciences Centre, ISPA–Instituto Universitário, Rua Jardim do Tabaco Lisbon, Portugal; 2 IBAP–Instituto da Biodiversidade e Áreas Protegidas, Avenida Dom Settimio Arturo Ferrazzetta, Bissau, Guinea-Bissau; 3 CESAM, Departamento de Biologia Animal, Faculdade de Ciências, Universidade de Lisboa, Campo Grande, Lisbon, Portugal; 4 Coastal Planning Office, Bairro de Belém, Bissau, Guinea-Bissau; 5 Centre for Ecology and Conservation, University of Exeter, Penryn, Cornwall, United Kingdom; 6 BirdLife International, Africa Partnership Secretariat, Dakar Fann, Senegal; 7 Bioinsight, Lda. Rua Antero de Quental, Odivelas, Portugal; University of Lleida, SPAIN

## Abstract

Vultures constitute an important functional group in many ecosystems, providing crucial ecosystem services both in natural and humanized environments. These scavengers are facing massive declines worldwide, but in several African countries virtually nothing is known on populations’ status and threats, hampering the development of adequate conservation strategies. In Guinea-Bissau, globally important populations of Hooded *Necrosyrtes monachus* and African white-backed vultures *Gyps africanus* were recently reported. Using the country as a study area, we aim to characterize human-vulture interactions in West Africa applying a multidisciplinary approach. We assessed the status and distribution of vulture populations using data from 1711 km of roadside transects, examined predictors of their distribution, and produced a nationwide population estimate for the Hooded Vulture, using an innovative method based on the relationship between the size of human population in settlements and vulture numbers. We conducted 47 stakeholder interviews to assess perceived roles played by vultures, and to investigate potential anthropogenic threats. Hooded vultures were strongly associated with high human population densities, whereas no relation was found between African white-backed and Rüppell’s vultures and any of the tested predictors, which included cattle density, precipitation and Normalized Difference Vegetation Index, among others. We estimate a national population of 43347 Hooded vultures, the largest population reported in the species range. Respondents were generally aware of the services provided by vultures, especially waste and carcass removal, including in urban areas. Hunting for witchcraft and traditional medicine was the most frequently recognised threat, while poisoning was ranked as having the highest impact. We hypothesise that poisoning-related mortality may be affecting African white-backed and Rüppell’s vultures’ distribution and explain their scarcity in apparently highly suitable habitats. Our results suggest a mutualistic rather than a commensalistic relationship between vultures and humans, with important implications for designing and implementing conservation strategies.

## Introduction

Scavenger vertebrates constitute an important functional group in many ecosystems, playing a critical role in many ecological processes [1]. Vultures are the only vertebrates that are obligate scavengers, being almost exclusively dependent on carrion for food [2–4]. Because they have evolved so many morphological and physiological adaptations, this group of birds plays a unique role in the functionality of some terrestrial ecosystems [2,5–8].

The importance of vultures on ecological processes is complex, but here we highlight two aspects: first, as nature’s most successful scavengers, they contribute greatly in the nutrient cycling dynamics by increasing the decomposition rate of carcasses and positively affecting transmission of nutrients to the soil [9,10]. This has a chain-effect that is important in maintaining biodiversity [11]. Second, some species also provide important ecosystem services in humanised areas, like the disposal of organic waste, the control (through competition) of opportunistic mammalian scavengers and of disease transmission, among many others [12,13].

Such specialized birds are particularly vulnerable to extinction and vulture populations are facing massive declines in most ecosystems [3,7,14]. Several causes have been reported throughout the world [6], with intentional poisoning and sanitary regulations in Europe [15,16] and poisoning by diclofenac residuals in livestock carcasses in Asia [17–20] being the most outstanding in those continents. In Africa multiple decline causes have been described; unintentional and intentional poisoning, intensive use for traditional medicine and sorcery, and hunting for food are the threats with the greatest reported impact on vultures [6,12,21–39]. As key-stone species, vulture declines have a wide range of impacts, including effects on human health, disposal economic costs to local communities, cultural and religious values and several other biodiversity impacts, including changes in the community structure (due to the decrease of the decomposition rate of carcasses) and in the composition of scavengers at carcasses and dumpsites (with increase of populations of opportunistic scavengers like canids and rodents, which are well-known disease reservoirs) [10,12,40].

The West African region has been reported to have one of the highest vulture population decline rates in the continent [3,41,42]. A recent survey of the birds of prey of Guinea-Bissau [43] found a high relative abundance of Hooded *Necrosyrtes monachus* and African white-backed vultures *Gyps africanus*, but other than that virtually nothing is known on the numbers, distribution and threats of these Critically Endangered species in that country. Also, understanding the type and magnitude of the interactions between vultures and local human populations and perceptions about these species is critical to outline effective conservation strategies and thus maintain ecosystem services these species provide in some West African urban areas.

In this study, we aim to investigate and characterize human-vulture interactions in West Africa, using Guinea-Bissau as a study area, a country still holding internationally important populations of vultures. We first assessed the distribution and abundance of Critically Endangered vulture populations in the country, mapping the distribution of the most abundant species, studying the predictors of their distribution, and delivering nationwide population estimates using an innovative and simple method based on a relationship between the size of human populations and vulture numbers, extrapolated to the whole country. Then, we also explored social aspects of vulture conservation to assess perceived roles played by vultures and the perceived importance of their conservation, and identify anthropogenic activities potentially affecting vultures in the study area. The social aspects of vulture conservation have also been particularly under-researched worldwide (but see [25,44,45]) but are crucial if we aim to effectively identify, design and implement robust conservation interventions and preserve the associated ecosystem services.

## Methods

### Study area

The study was conducted from the 19th February to the 28th April, and from the 7th November to the 7th December 2016 in Guinea-Bissau, a small (36125 km^2^) West African country with a humid tropical climate with rainy (from May to October) and dry (from November to April) seasons [46]. Human population in 2016 was estimated at ca. 1.55 million, with about 42.6% living in urban areas [47]. As one of the poorest countries in the world [48] and home to more than 30 ethnic groups, households are highly dependent on natural resource use for subsistence while economic activities are still rather underdeveloped [49].

**Fig 1:**
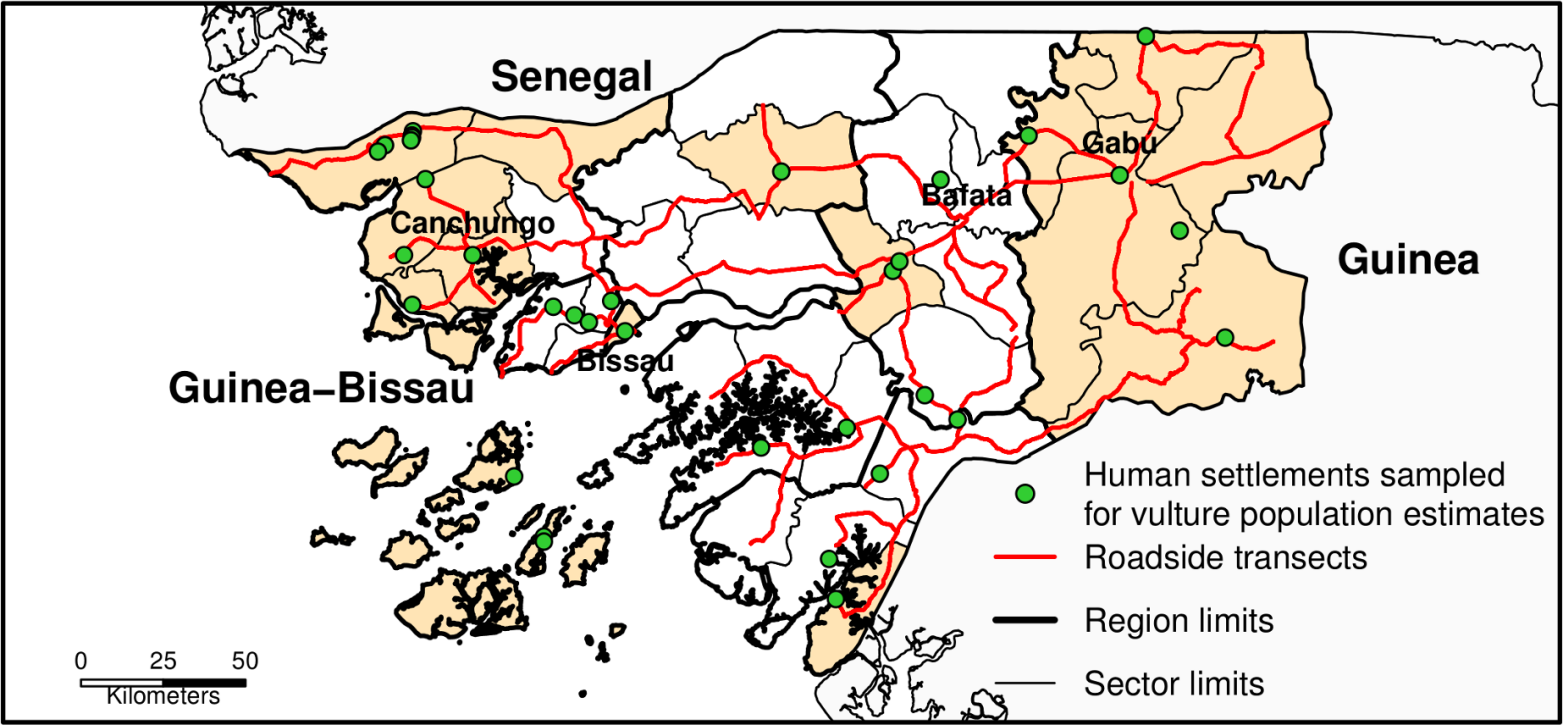
Map of the study area. Areas without red lines correspond to Sectors (an administrative division within Guinea-Bissau) that were not sampled for the study of vulture distribution and abundance (i.e. no road surveys were conducted). Orange areas are Sectors where interviews were carried out for the social surveying component of our study. The four major cities of the country are represented by their names in black writing.

To map the distribution and investigate the potential drivers of distribution and abundance of the most abundant Critically Endangered vulture species, we sampled 32 (out of 37) Sectors, an administrative division within Guinea-Bissau, which are then grouped in seven (out of eight) Regions; the five remaining Sectors were not sampled due to logistic constraints (i.e. absence of roads and time/budgetary limitations). To estimate the abundance of Hooded vultures in the country, we sampled 34 human settlements distributed all over the country, which were of different sizes in order to obtain a good representation of the study area characteristics; and to study the social aspects of vulture conservation, we conducted interviews in 17 Sectors, selected to represent as much as possible of the country’s ethnic and cultural diversity while minding logistic and budgetary constraints (Fig 1). Permission to conduct the study in the country was issued by the public Institute of Biodiversity and Protected Areas, which also provided full and unrestricted access to all protected areas. Permission to conduct the study in the settlements was obtained in every location from local governmental authorities (Administrators, the governmental leaders of each Sector, based at the capital of the Sector), local government representatives (“Comité de Estado”, the representatives of the Administrators in each settlement of the Sector) and traditional leaders, to which we always payed visit beforehand in order to introduce ourselves and the project; as these individuals were often also interviewed for this study, names were not recorded to protect anonymity.

### Mapping of the distribution and abundance of vultures

The relative abundance of vultures in Guinea-Bissau was assessed using road surveys by car (1711 km of transects; see full methodological details in [43]). Using data from those surveys, we calculated encounter rates in each Sector (expressed as birds per 100 km of transect) for the most abundant Critically Endangered vulture species, which were used to give a portrait of the relative abundance of each target species in the country. We aggregated all African white-backed and Rüppell’s *Gyps rueppelli* vulture’ detections in one group (*Gyps* spp.) because we were often not able to distinguish between juveniles of these species. Rüppell’s vultures are rare and therefore *Gyps* spp. refers mostly to African white-backed vultures. In the Bijagós archipelago road surveys were not carried out due to the absence of roads, but we include anecdotal observations collected during the course of other projects.

### Predictors of distribution and abundance

We used General Linear Models (GLM) to investigate the relationship between the encounter rates of each species in each Sector, and the following explanatory variables: average Normalized Difference Vegetation Index (NDVI) of the three driest and of the three wettest months, mean annual temperature, average annual precipitation, cattle density (heads per km2, including all types of cattle except for poultry), human population density (people per km2) and Global Burned Area (GBA; MODIS MCD45A1 v). The NDVI differentiated open areas (which are drier) from more dense habitats, and Gyps vulture movements have been reported to be related to different NDVI values [50]. The GBA, a monthly Level-3 gridded 500-m MODIS product containing per-pixel burning and quality information [51], was used as a proxy for suitable foraging areas for cattle and, thus food availability for vultures. Climatic variables were obtained from WorldClim (http://www.worldclim.org/; [52]). Cattle density was obtained from Region scale total cattle counts in the country [53], and then expressed as density at Sector level. Human population data derived from counts during national population census were also entered in the model as densities per Sector [54].

Variable selection followed a stepwise approach, based on Akaike Information Criterion (AIC) scores. When the model with lowest AIC included non-significant variables, we continued the stepwise procedure until only significant variables were left.

### Distribution of vultures in habitat classes

During roadside transects we registered, whenever possible, the habitat associated with each observation (within a 30-meter radius from the bird). We calculated the global proportion of birds of each species that were perched or flying over a certain type of habitat in the moment of detection. Habitats were grouped in 9 classes (Table 1).

**Table 1:** Habitat classes created for the analysis of the distribution of Hooded and *Gyps spp*. vultures over habitats.

Habitat class	Description
**“Bolanhas”**	Rice fields in wet and saline environments.
**Cultivations**	Plateau rice fields and gardens.
**Forests**	All kind of natural forests, including palm tree forests.
**Human Settlements**	All type of settlements with permanent or periodic presence of people.
**“Lalas”**	Vast low ground areas flooded periodically, with grassy vegetation.
**Mixed**	Areas with both natural and anthropogenic environments.
**Orchards**	Mainly cashew trees, with also some mango trees.
**Savannahs**	Sudan-Guinean savannahs, including wooded savannas.
**Wetlands**	Permanent or almost permanent humid habitats, including mangrove and rivers (both salty and freshwater rivers).
**NI**	Non-identified habitat (when habitat was not visible from the road).

### Nationwide population estimates

Given the strong connection of Hooded vultures to humans (we know of no roosts outside human settlements), we estimated the population of this species by sampling 34 human settlements of different sizes, during periods of low activity of these vultures to avoid double-counts. We used two different and complementary sampling methods, according to the type of human settlement:

#### (1) Density estimates using strip transects in large cities

We conducted transect counts using a bicycle, between 7:00 and 8:20 and between 17:30 and 19:20, when most vultures were perched or foraging on the ground [43]. Sampling design was based on literature recommendations for line and strip transects [55–57]. The surveys were conducted by one observer, which counted every perched Hooded vulture seen during transects. Perpendicular distances of each bird to the transect line were precisely measured with a laser rangefinder. This method was only used in the two largest human settlements of the country, Bissau and Gabú (82.9 and 8.7 km^2^ of total area, respectively; Fig 1). In Bissau, we conducted 14 transects along 71.4 km of roads and streets and in Gabú we conducted 3 transects along 16.9 km.

We estimated the density of Hooded vultures (birds per km^2^) as the number of individuals within 60 m (30 m on each side) of the transect, using the strip transect method [55–58] and then, extrapolated the number of birds for the area of each city (see details on strip width selection in S1). Since in the calculations we only accounted for perched birds, we applied a correction factor of 1.12 for Hooded vultures flying during low activity periods, based on daily activity pattern counts [43] (see details in SI1).

#### (2) Complete counts of roosting Hooded vultures in other human settlements

In villages and towns, we conducted counts by car (using three to four observers) in the early morning and late afternoon, obtaining a minimum number of Hooded vultures roosting in each sampled site. During these counts, we slowly crossed roads and pathways through all the settlement and counted all individuals seen perched or flying. Using this method, we sampled 17 villages, 14 small towns and 1 larger town (Fig 1).

We used Generalised Linear Models (with quasi-Poisson error distribution) to relate the number of Hooded vultures with the (natural log) number of people in settlements as obtained in the national human population census. This model was highly significant (see Results) and therefore was used to predict the number of Hooded vultures in each (non-surveyed) settlement in Guinea-Bissau. These individual estimates were then summed up to deliver an estimate for the national population. The cities of Bissau and Gabú were not used in the models, as they were clearly outliers in terms of both human population and number of vultures, and had an undue influence on the parameter estimation. The estimate of the total number of vultures and the corresponding confidence interval were obtained by a bootstrapping technique. We generated 1000 Generalised Linear Models based on bootstrap samples (with replacement) of the 32 settlements sampled and their corresponding vulture counts. Each model was used to deliver a single prediction of the total number of vulture as the sum of the estimated value for each settlement of the national census. We then calculated the median of these values as well as the confidence intervals, which were estimated by the percentile method [59] as the 0.025 and 0.975 quantiles of the distribution of the 1000 replicates. The counts made in Bissau and Gabú using the strip transects were then added to these values.

### Exploring social aspects of vulture conservation

Seventy-four stakeholders were interviewed in 47 interviews (39 of which were individual interviews and 8 were group interviews involving 35 stakeholders) throughout the country, targeting people involved in activities potentially related to the conservation, monitoring and exploitation (e.g. harvest for food, traditional medicine, poisoning) of vultures in Guinea-Bissau. Snowball sampling was used to select participants in the survey, using recommendations from interviewees to establish contact with others most relevant to the study. This purposive sampling approach is suitable for identifying stakeholders and capturing the widest range of perspectives, while avoiding potential biases due to researchers’ perceptions about the system [60]. Key stakeholders interviewed included: local authorities, both governmental (n = 4) and religious/cultural (n = 11), slaughterhouse employees (n = 1), livestock farmers (n = 8), farmers (n = 7), veterinaries (n = 8), conservation and sustainable development agents (n = 5), and witchcrafters/traditional healers (n = 11). Only one (group) interview happened to include three female respondents, which somewhat reflects their smaller involvement in the targeted professional groups in the area. Age class of respondents was distributed as follows: three interviews to an age range of 20–34, 10 to 35–49, 15 to 50–64 and eight to 65 or more (see detailed description of the 74 stakeholders interviewed in S1 Table).

Research was approved by the University of Exeter Ethics Committee (Ref. 2016/1422). All individual interviews were carried out in private, and group interviews were conducted only when an individual approach was not possible. Consent for participation was obtained before each discussion and information was kept anonymous. Written consent was not required because no personal data or personal identifiers were collected. It was made clear that consent was implied from participating in the survey. Interviews were conducted by MH and HM in Guinea-Bissau creole. Seventy-three (out of 74 study participants) were presented with questions about their role within the study system, and closed and open-ended questions to gather information about their personal experiences and perceptions (e.g. perceived trends of Hooded and White-backed vulture populations), followed by two exercises aiming to identify issues potentially affecting the conservation of vultures in Guinea-Bissau (see full questionnaire in S2 Appendix):

#### (1) Perceived roles played by vultures

Assessment of perceived services (and potential disservices) provided by vultures was undertaken by asking respondents about specific processes (i.e. food, sorcery, clean-up, cultural) that could affect people (both “themselves” and “other people”) in Guinea Bissau. The Millennium Ecosystem Assessment framework [61] was used as guideline for categorization of ecosystem services. An indicative assessment of the nature (positive, negative or indifferent) of the general perception of each respondent about the role of vultures for humans was also undertaken by the interviewer based on the respondents’ answers to the questions about specific processes. When this assessment was not clear from the previous answers, the respondents were directly asked about the nature of their perception. We also probed for reasons for this general perception.

#### (2) Threat analysis and ranking exercise

Respondents were asked to list factors that in their opinion are negatively affecting the conservation of vultures in Guinea-Bissau, distinguishing between direct threats and underlying factors (indirect threats). Direct threats are factors that can directly cause the reduction or loss of vultures (e.g. poaching), whilst indirect threats are factors that contribute to the presence or persistence of direct threats (e.g. weak law enforcement). Belief-based use (for religious and/or cultural rituals) and unintentional and intentional poisoning were assessed with directed questions whenever the respondents made no reference to it. A ranking exercise to assess the spatial scope, severity (level of impact) and reversibility (capacity to recover) of each of the identified threats was also undertaken following the methods in IUCN and ICCN (2012) [62] (see Table 2). Average values were obtained for each threat and a final score (resulting from the sum of the average values of spatial scope and severity) was used to rank threats by importance.

**Table 2:** Criteria used for ranking spatial scope, severity and reversibility in the threat-ranking exercise*.

Value	Spatial scope	Severity (level of impact)	Reversibility (capacity to recover)
0	Absent	No impact or minimal	Easily reversible
1	< 25%	Moderately degrades	Reversible if there is enough commitment
3	25–75%	Seriously degrades	Reversible but with great difficulty
5	> 75%	Completely destroys or eliminates	Not reversible

Spatial scope: Proportion of the vulture's range likely to be negatively impacted by the direct and indirect threats; Severity: Level of impact of the direct and indirect threats; Reversibility: Capacity to recover from the effects of the direct and indirect threats.

*Adapted from IUCN and ICCN (2012).

The questionnaire included a separate section directed only at veterinaries (n = 8 interviews to 10 stakeholders) and livestock-farmers (only those personally delivering veterinary care to their cattle, n = 2 individual interviews) to assess the potential presence and use of Non-Steroidal Anti-inflammatory Drugs (NSAID’s) such as Diclofenac (see separate section of the questionnaire in S3 Appendix). One of the 74 study participants was only interviewed for this purpose.

All analysis were carried out in R 3.3.0 [63]

## Results

### Distribution and abundance of vultures

**Fig 2:**
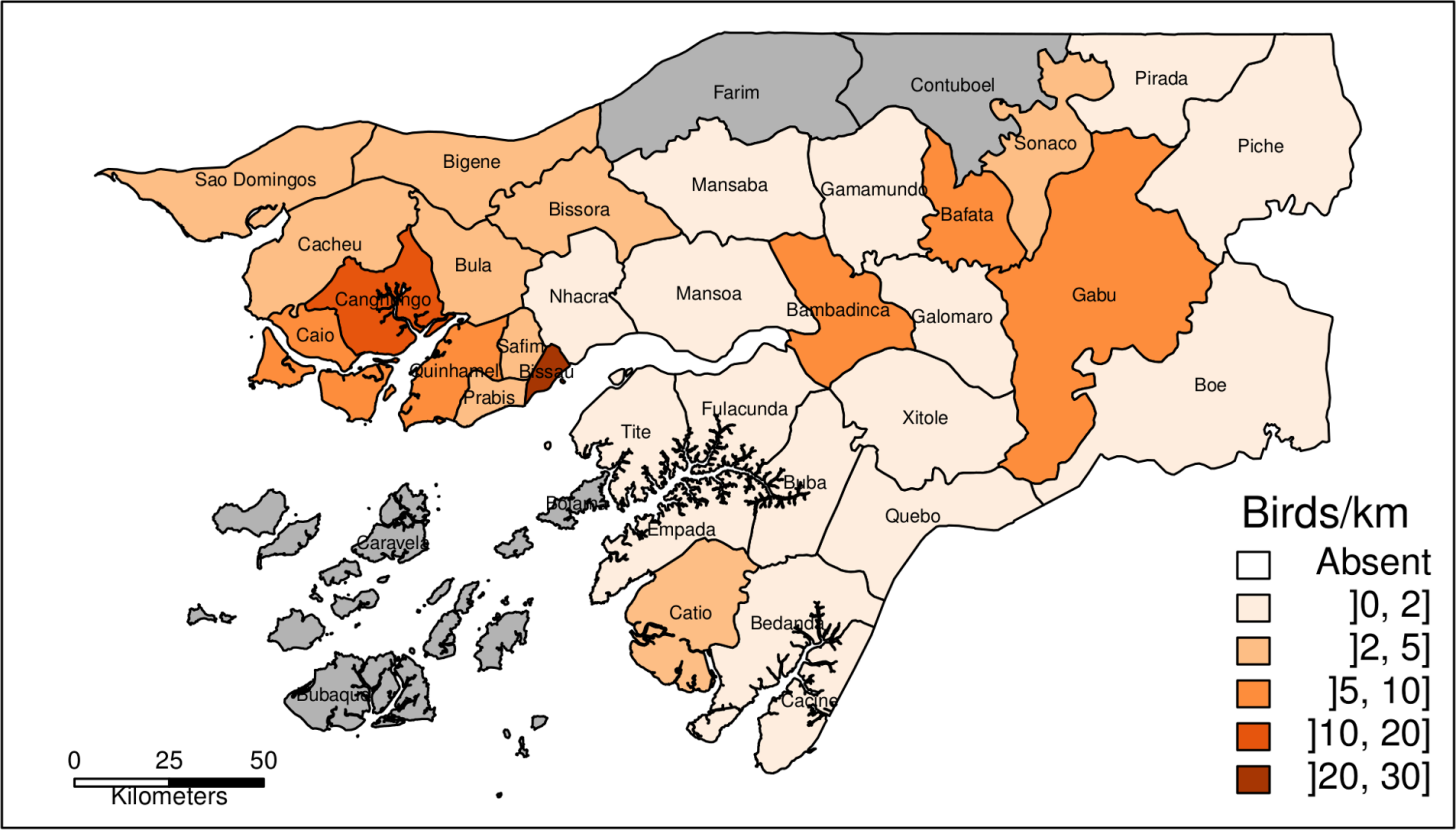
Abundance of Hooded vultures. Abundance of Hooded vultures *Necrosyrtes monachus* in sampled Sectors of Guinea-Bissau obtained from road survey transects. Grey areas represent Sectors that were not sampled (yet Hooded vultures are present and widespread in the Bijagós archipelago; authors pers. obs.).

**Fig 3:**
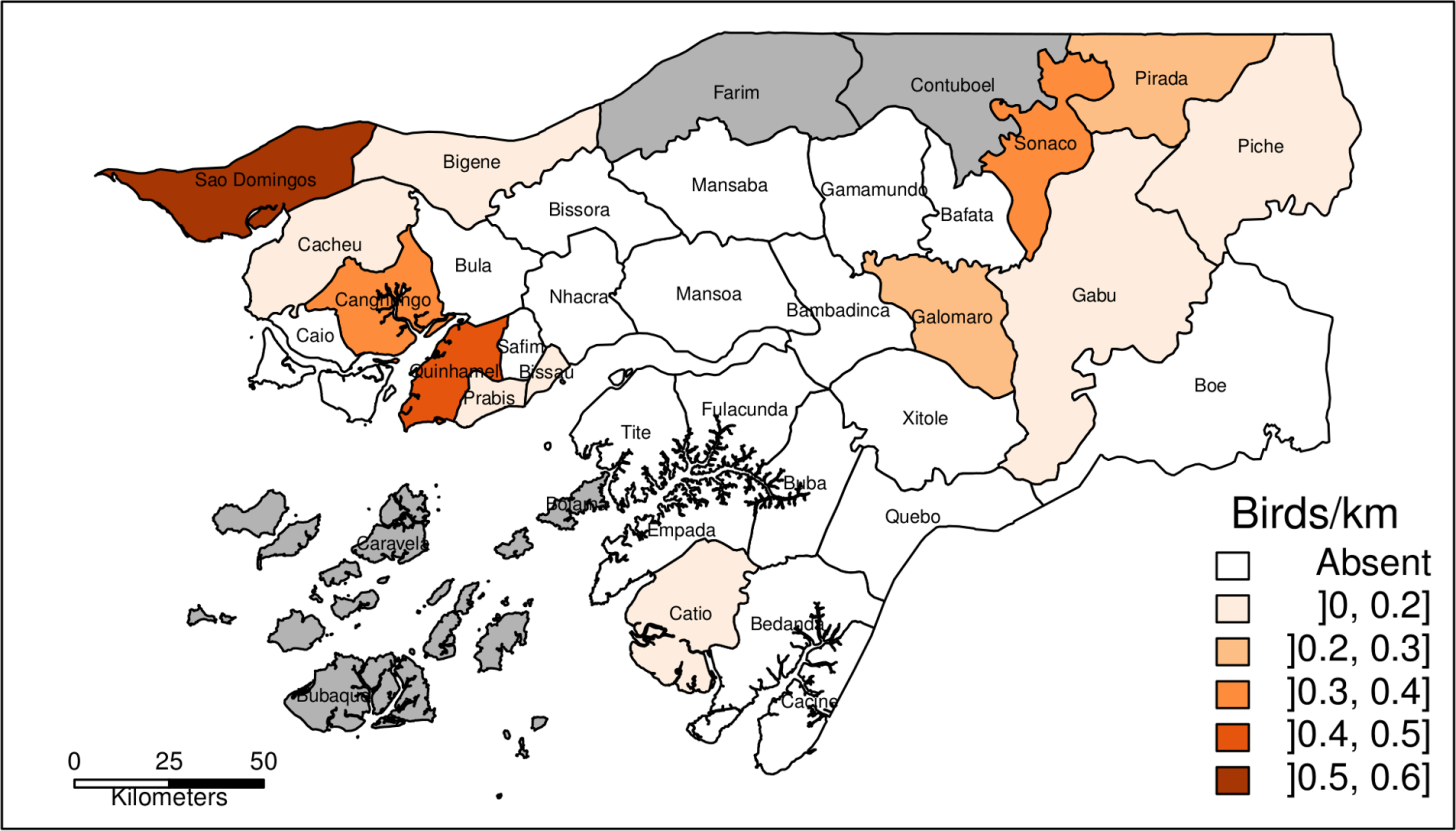
Abundance of African white-backed and Rüppell’s vultures. Abundance of *Gyps spp*. in sampled Sectors of Guinea-Bissau obtained from road survey transects. *Gyps spp*. consist mostly of African white-backed vultures. Grey areas represent Sectors that were not sampled (*Gyps spp*. were never observed in the Bijagós archipelago; authors pers. obs.).

Hooded vultures were present in all Sectors sampled, being noticeably more abundant in Bissau (Fig 2), and were also present in almost every inhabited island of the Bijagós archipelago (authors pers. obs.). The lowest abundance was recorded in the eastern part of the country (Boé, 2 birds seen in 103.7 km of transect). African white-backed and/or Rüppell’s vultures (*Gyps spp*.) were detected in 13 Sectors, with highest abundance in the northern sectors (São Domingos, Quinhamel and Canchungo, Fig 3). In the south, there were almost no *Gyps spp*. (only 4 birds detected south of the Geba river, in c. 760 km). Since 98% of the sightings of Gyps vultures with confirmed identification were African white-backed vultures, the abundance distribution in Fig 3 may also be used to represent this species (see raw data in S2 Table).

### Predictors of distribution and abundance

According to the GLM results, Hooded vulture abundance was significantly related to (natural log) human population density, whereas all other variables were not significant (p-values>0.05, Table 3). *Gyps spp*. showed no significant relationship with any of the selected explanatory variables.

**Table 3:** General linear models used to evaluate the effect of predictors on the distribution of Hooded vultures and results of the final models. AIC values were used to select the best model in a forward stepwise method.

Model selection			df	AIC
1 NDVI driest quarter+NDVI wettest quarter+ Mean ann. temp+ Mean ann. prec+Cattle+ Hum. dens +Prop._burn. area			7	63.38
2 NDVI driest quarter+NDVI wettest quarter+Mean ann. prec+Cattle+Hum. pop. dens+Prop._burn. area			6	61.4
3 NDVI driest quarter+NDVI wettest quarter+Mean ann. prec+Cattle+Hum. pop. dens			5	59.43
4 NDVI driest quarter+NDVI wettest quarter+Mean ann. prec+Hum. pop. dens			4	57.94
5 NDVI wettest quarter+Mean ann. prec+Hum. pop. dens			3	56.84
6 NDVI wettest quarter+Hum. pop. dens			2	55.78
7 Hum. pop. dens			1	54.62
**Final Model**	**Estimate**	**SE**	**t-value**	**p-value**
Intercept	1.29693	0.62954	2.06	0.0485
Human population density	0.02738	0.01074	2.55	0.0163

### Distribution of vultures in habitat classes

**Fig 4:**
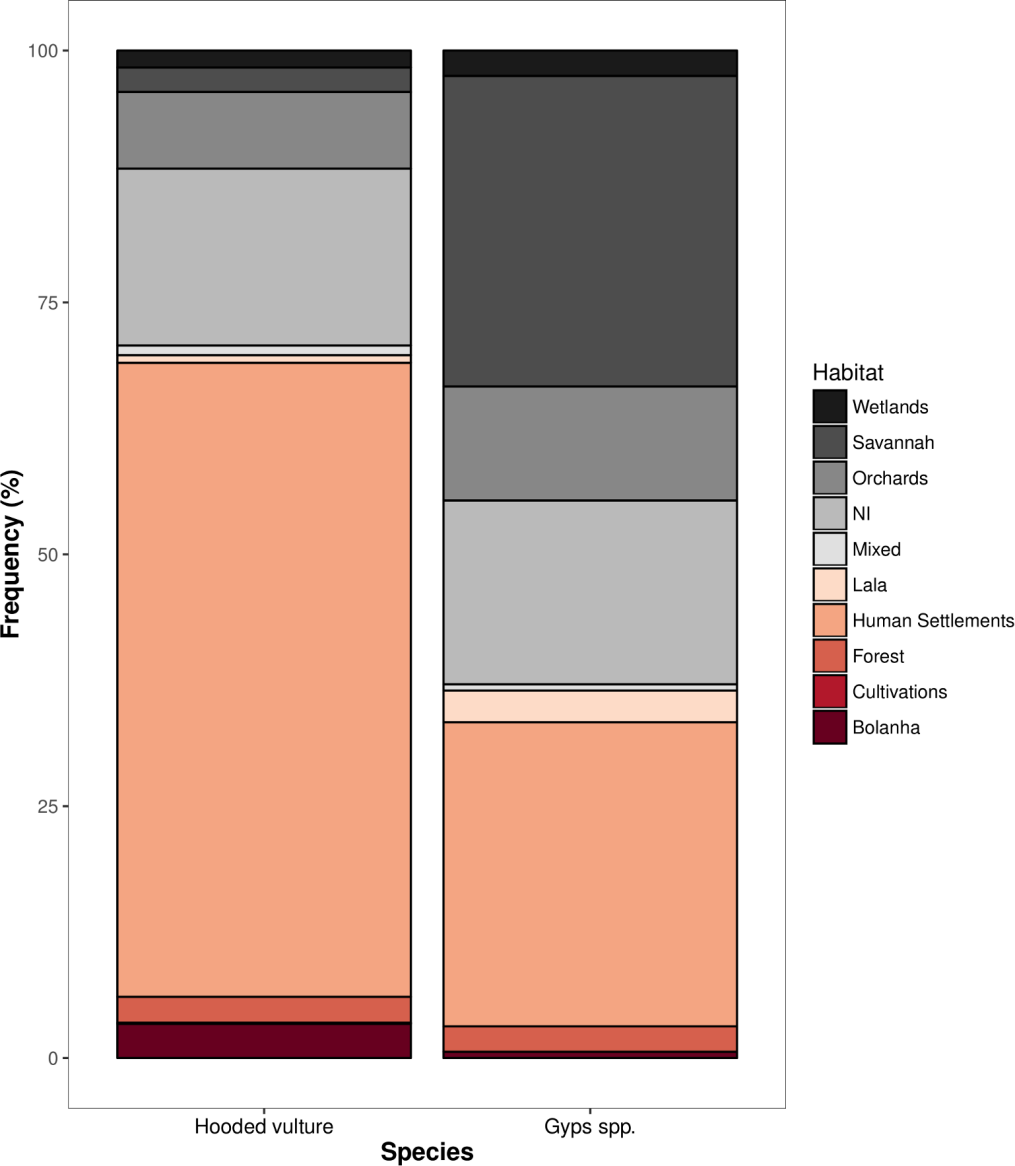
Distribution over habitats of most abundant vulture species. Distribution over habitats of most abundant vulture species during transect surveys in Guinea-Bissau mainland. (NI–Non-identified habitat). *Gyps spp*. consist mostly of African white-backed vultures.

Both Hooded vultures (n = 4360) and *Gyps spp*. (n = 159) were detected frequently in human settlement areas (63 and 30%, respectively; Fig 4), although *Gyps spp*. were slightly more associated with savannahs (31%; see raw data in S3 Table).

### Nationwide population estimates

#### (1) Density estimates using strip transects in large cities

Bicycle transects in the city of Bissau yielded a total of 1071 Hooded vultures, with a density of 76.6 perched birds/km^2^, resulting in an estimated total of 7117 individuals (after applying the correction factor for birds flying, see details in SI1). In Gabú we estimated a density of 101.8 birds/km^2^, resulting in a total abundance estimate of 991 Hooded vultures (see transect raw data in S4 Table).

#### (2) Complete counts of roosting Hooded vultures in human settlements

We counted a total of 1243 Hooded vultures in 32 human settlements during low activity periods, with values ranging from 0 to 201 individuals. Among these, 10 settlements (all with less than 600 people) had no vultures. Nonetheless, vultures were also present in settlements with less than 600 people (see raw data in S5 Table). The number of Hooded vultures counted was significantly related to the (natural log) number of people in each settlement sampled (Generalised Linear Models with quasi-Poisson error distribution, effect of number of people in settlement: 0.713±0.173, t = 4.13, P<0.001, intercept: -1.639±1.397, t = -1.17, P = 0.249, ANOVA, F_1,30_ = 21.81, P<0.001). Using this model, we predicted the number of vultures in each of the 4422 human settlements identified in the country (mainland and Bijagós archipelago included; [54]). After summing all these individual estimates (and bootstrapping, see methods section), we estimated the number of Hooded vultures in Guinea-Bissau (excluding Bissau and Gabú) at 35239 birds (median value, 95% confidence intervals = [14558–80465]). Adding the results from Bissau and Gabú to the above estimate we obtain a total of 43347 Hooded vultures in all of Guinea-Bissau territory (95% confidence interval = [22666–85573] birds).

### Exploring social aspects of vulture conservation

When asked about the importance of vultures, most interviews (66%) indicated that respondents perceived vultures to generally have a positive role, while in 21.3% respondents expressed a negative opinion about them and in 10.6% people claimed to be indifferent (n = 46 interviews). The most frequent reasons presented for the positive perceptions involved recognition that vultures provide useful services and never cause harm to people or other animals (Table 4). Negative perceptions were mostly justified by the fear of vultures being vectors of transmission of diseases and/or because they were considered “annoying” by some meat sellers and livestock-farmers (Table 4).

**Table 4:** Perceived positive or negative aspects of vultures according to stakeholders. Perceived positive or negative aspects of vultures according to stakeholders in Guinea-Bissau, with categories expressed as percentages and with example quotes. Each interview could refer more than one reason (46 and 13 references to positive and negative reasons, respectively).

Justification	Numb. of references	Quotes from interviews*
**Positive aspects (n = 31 interviews)**		
Useful (for clean-up, to find lost cattle or people, for witchcraft and traditional medicine)	20	“Vultures clean waste from our villages” “They help cattle owners find their lost cattle when it gets lost in the forest or dies somewhere” “Once there was a boy that died from a Buffalo attack and his corpse was only found thanks to vultures” “Vultures act like the Municipality of the cities, cleaning up the garbage from the streets” “There are certain human ailments people say that can be cured using vultures in rituals. They say you can cure lepra if you eat vulture meat.” “They say if you eat vulture meat you cannot be detained by government services or go to jail or if you go to jail you get released right away”
Harmless	17	“Vultures do not attack any living beings, they just go for things that are already dead”; “Vultures do not create conflicts with people”
Belief-based (religious and traditional/cultural beliefs)	8	“Vultures are like divine deities, they say if you kill one you will catch some sort of disease” “Vultures are creatures of God, they do not harm anyone and we should not kill them because they were sent by God” “It is very difficult for people to kill vultures because they are afraid of what might happen if they do, as they say vultures have spiritual protection”
Cultural	1	“Vultures are the symbol of a matriarchal family line in the Bijagós ethnic group”
**Negative aspects (n = 10)**		
Spreading disease	6	“We do not have nothing against vultures, but we are not sure if they are not carrying diseases and passing them to us when they come near people and in our slaughterhouses” “Vultures travel far and carry diseases with them from one place to another” “We think vultures eat cows that died with diseases and they can transmit that disease to us. We think there are too many vultures nowadays”
Annoying	5	“Vultures steal meat and when you are not looking they poo on the meat” “It is an animal that annoys’ us livestock herders, when there is a dead animal or a newly born cow, they come to feed on it. Before they were afraid of men but now they are hard to send away” “Vultures are like a domestic animal that is annoying for the community”
Useless	1	“I do not see them as good animals, they are not edible, they are useless”
Disgust	1	“People say that Muslims are disgusted by vultures because they eat human faeces. It is an animal that feeds in dirty places, it is not clean, and it feeds on microbes”

*Quotes from interviews translated by MH from Guinea-Bissau creole.

**Fig 5:**
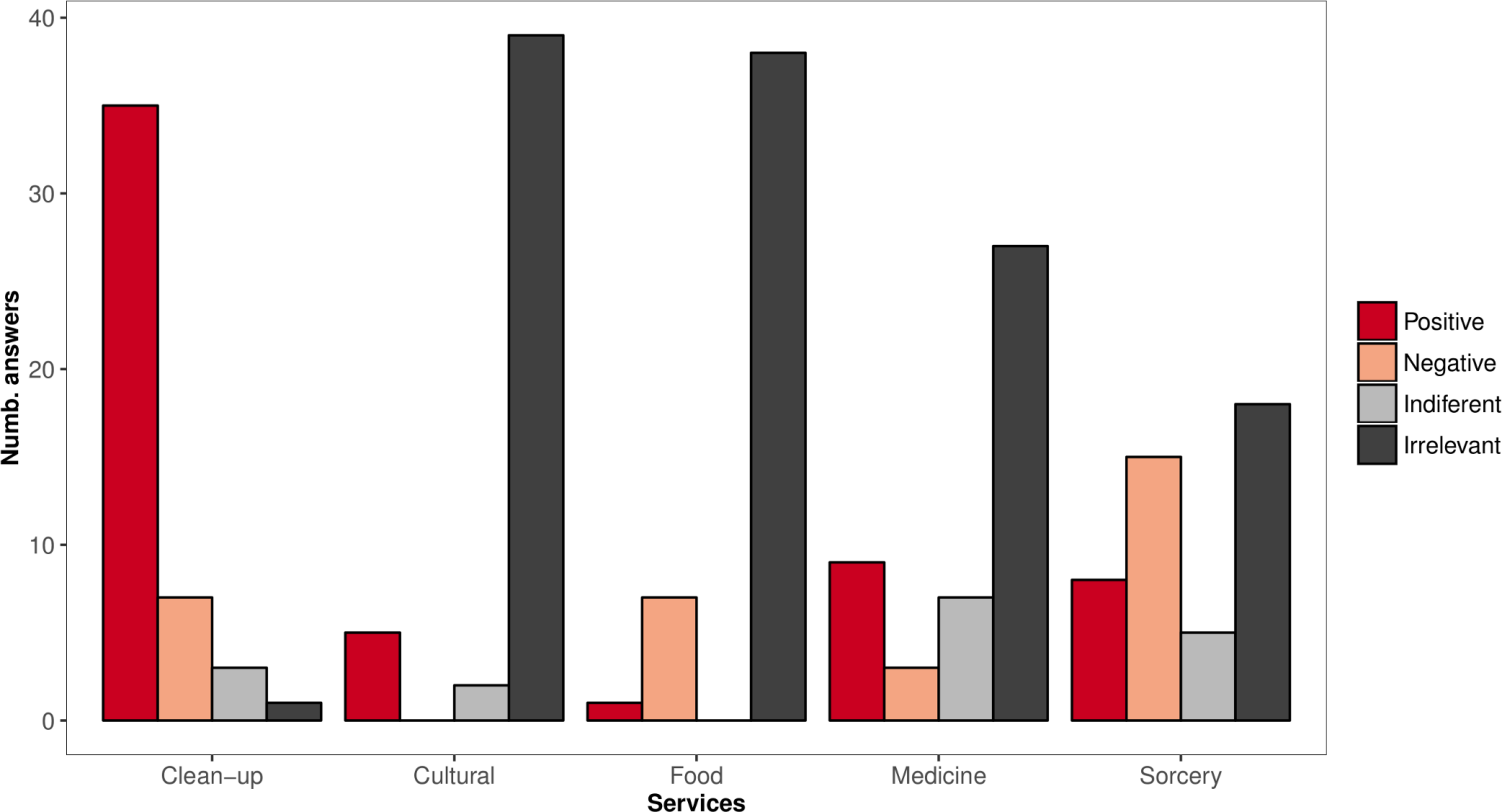
Perceived roles played by vultures in Guinea Bissau according to stakeholders. Perceived roles played by vultures in Guinea Bissau according to stakeholders, showing the frequency (in number of answers; n = 46) of interviews to respondents that regarded vultures as positive, negative, indifferent or irrelevant for each of the referred services.

In most of the 46 interviews where 73 respondents were asked about perceived roles of vultures in Guinea Bissau, vultures were reported to be irrelevant for local culture (85%) and as food source (83%), and most interviews (59%) also indicated that these respondents were unaware of the use of vultures for traditional medicine (Fig 5). However, in most interviews (76%), respondents recognized that vultures provide a positive clean-up service of urban waste and carcasses of dead animals. Most interviews (61%) indicated that respondents were also aware of the use of vultures for witchcraft, with 33% regarding this as negative. Nonetheless, a relevant part (28%) reported this practice as positive or respondents did not oppose to it (Fig 5).

Of the 46 interviews in which 73 respondents were asked about perceived trends of Hooded vulture numbers in their area over the last 10 years, 41% believed they were increasing, 26% said they were decreasing, and 24% believed numbers were stable (9% did not report an opinion or did not know). When asked about perceived trends about *Gyps spp*. abundance, most interviews (37%) indicated that respondents never or seldom saw/recognized the species, while in 30% respondents believed they were decreasing, 11% reported they were stable and 9% stated an increase (13% did not have an opinion).

**Fig 6:**
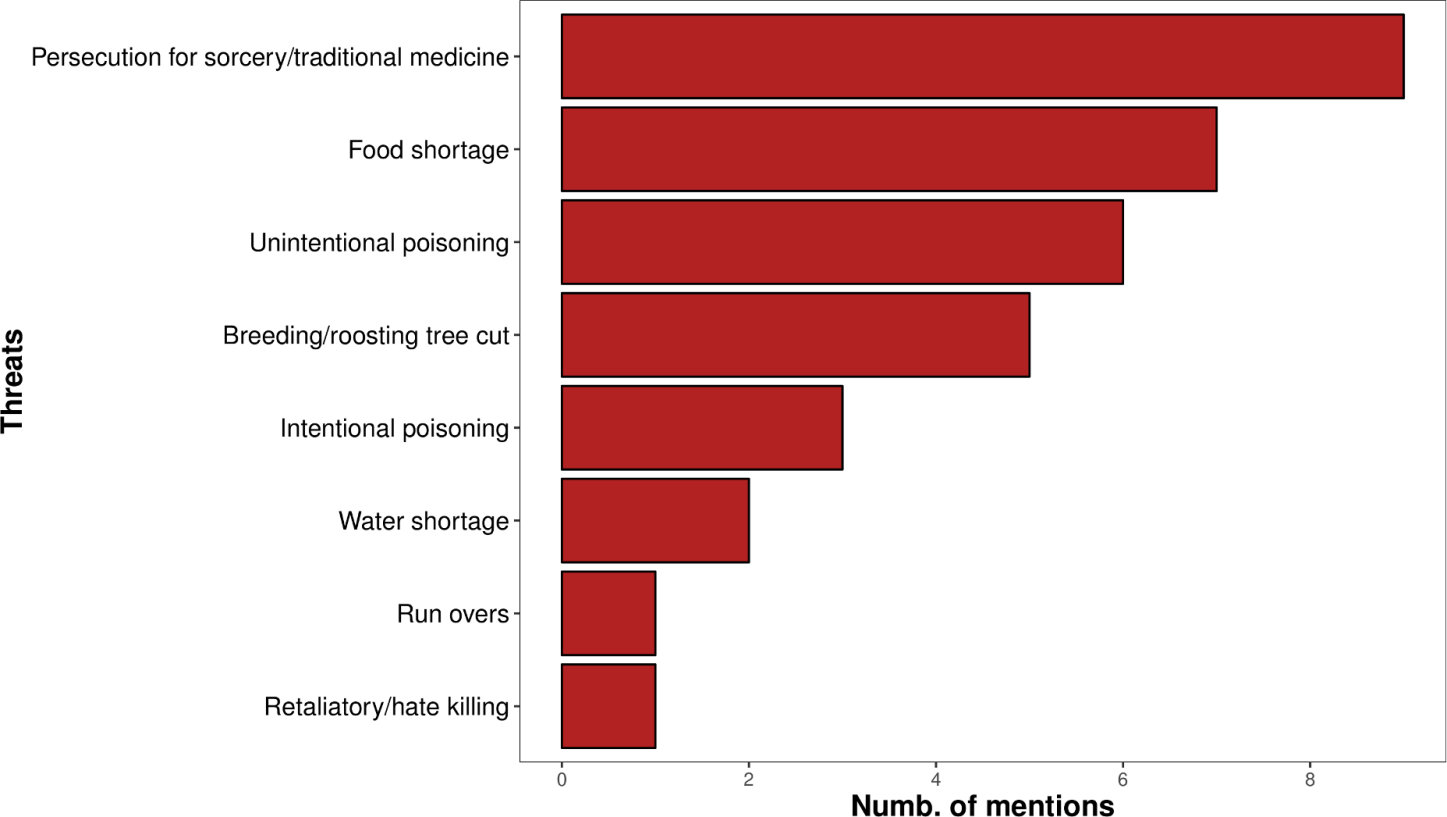
Threat analysis exercise. Threat analysis exercise, showing the number of mentions of each threat. In each interview, respondents could mention more than one threat, thus total exceeds sample size/number of interviews (n = 23).

Half (50%) of the 46 interviews in which 73 respondents were engaged in the threat analysis exercise indicated that respondents believed there were no threats to vulture populations in the country. Among the remaining 23 interviews, eight different threats were mentioned, of which the persecution for sorcery and traditional medicine were the most frequent (Fig 6).

In the threat ranking exercise, unintentional and intentional poisoning were the direct threats ranked with the highest scores. Similarly, breeding and roosting tree cut was the indirect threat with the highest score, and also considered to be the less easily reversible, along with food shortage and unintentional poisoning (Table 5).

**Table 5:** Mean values of the threat ranking exercise (n = 23).

Threats mentioned by stakeholders	Spatial scope	Severity	Score	Reversibility
**Direct threats**				
Unintentional poisoning	4	3.7	7.7	2.7
Intentional poisoning	3.7	2.3	6.0	1.7
Retaliatory/hate killing	5	0	5	0
Persecution for witchcraft/traditional medicine	3.4	0.7	4.1	2.4
Run overs	1	0	1	0
**Indirect threats**				
Breeding/roosting tree cut	4.2	2.6	6.8	3.8
Food shortage	3.3	2.1	5.4	3.3
Water shortage	2	3	5	1

Higher values of spatial scope represent larger % of area affected); higher values of severity represent higher impact over vulture populations; and higher values of reversibility are interpreted as higher difficulty to reverse negative effects of the threats. Score = Spatial scope + Severity.

When asked directly about the most important target threats to vultures (i.e. intentional and indirect poisoning and persecution for body parts for witchcraft), witchcraft was the most recognized (79%), compared with unintentional (15%) and indirect (11%) poisoning (n = 46 interviews).

There were no mentions to past or present use of any NSAID’s, including diclofenac, during the 10 interviews to veterinaries and livestock-farmers.

### Discussion

This study confirms that Guinea-Bissau represents a stronghold for Hooded vultures and for African white-backed vultures, species which are present in large numbers and widely distributed across the country. Wild ungulates and other mammals have grown scarce in Guinea-Bissau, a general trend in most of West Africa [64,65], but nevertheless vultures are still abundant in that country, apparently relying on domestic animals (as has been the case for avian scavenger populations in Europe [66]) and human waste. Vultures are mostly present in densely populated areas, highlighting the importance of human-vulture interactions and the role of humans in the future of vulture populations in this African region. Likewise, key stakeholders seem to be mostly aware of the benefits generated by vultures, recognising their importance for them and for human society.

### Distribution and predictors of abundance: The human-vulture relationship

Our results clearly demonstrate the importance of humans in the distribution of Hooded vultures. These scavengers were more numerous and more frequently detected in areas with the highest congregations of people, where a great deal of rubbish and refuse are available. Bissau, Gabú, Bafatá and Canchungo, the four main urban centres of Guinea-Bissau, are the most extreme examples of this association, where Hooded vultures are highly conspicuous and can be found in any dumpsite and in large concentrations (up to several hundreds) in the main slaughterhouses of the cities. Similar results were obtained in a study conducted on Hooded vultures in Ghana, where the authors concluded that their presence is positively correlated with human presence, being strongly attracted to meat and waste production [45]. The lack of relationship between vulture numbers and broad habitat variables, such as NDVI or Global Burned Area may also suggest that Hooded vultures concentrate their foraging activity preferably in urban areas.

*Gyps spp*., mostly African white-backed, are still present in many parts of the country, and are even abundant in some Sectors in the northwest. However, these birds are very scarce or absent in the easternmost Sectors, where there are large extents of suitable habitat (savannah), including some protected areas with very low human densities. Some of these Sectors have the highest abundances of cattle in the country [53,67], providing a potential food source. We could not identify main drivers for the distribution of vultures belonging to *Gyps* genre. However, their scarcity in apparently suitable areas, may suggest a role for indirect poisoning. Anecdotal reports collected during interviews seem to suggest occasional poisoning campaigns organised by livestock herders and veterinaries as retaliation or prevention of attacks by large carnivores (such as hyenas and leopards). However, without further research, this remains a matter for speculation.

Nonetheless, the results of the distribution of vultures in different habitats provide a clear indication of a strong connection with human activities, particularly in Hooded vultures but also to a large extent in African white-backed vultures. Such high levels of association with people are probably due to the scarcity of wild fauna.

### Nationwide population estimates

Estimates of population size of Hooded vultures based on systematic work are rare. Only in Uganda, Tanzania, Mozambique, Ghana and in the Gambia there have been attempts (some very incomplete) to estimate the number of individuals [12,68–73], often based on counts at roost sites. In our study area, these methods would not have been adequate, mostly because Hooded vultures are very abundant and tend to roost scattered in many small groups, and it would be impossible to get a good coverage of thousands of roosts scattered all over the country.

There are limitations that need to be taken into account when interpreting national level estimates of population sizes based on counts on a sample of the study area, especially when considering abundant species with heterogenous distributions such as Hooded vultures. In this study, several measures were taken to minimize many of the possible sources of error [74]. These include using the same observers, standardizing the search effort within and among transects (i.e. speed and duration), conducting the counts only during the most adequate periods of the year (during the dry season, when trees have fewer leaves; during the breeding season, when birds are more active and conspicuous) and of the day (counting only during the most stable pre and post-roost periods [43]), and conducting counts in the same weather conditions. Moreover, our transect method also allowed us to minimize the uncertainty related to habitat variation, since there are no known Hooded vulture roosts outside human settlements in Guinea-Bissau, and we used two different approaches for more and for less urbanised settlements. We found a good correlation between vulture numbers and the size of human population, and therefore we explored this association to estimate the total number of vultures in the country. Like all estimates obtained by correlative models, this number should be considered as a preliminary approach to global numbers, and used in conjunction with their confidence intervals. Still, we suggest that this could be applied in other countries, with the necessary adaptations and methodological tests.

We believe our estimate of the number of Hooded vultures (43,347 birds) to be realistic. For example, during the three months spent in the field (including observations during road surveys) we actually counted 7175 Hooded vultures, directly confirming the abundance of the species. According to our results Guinea-Bissau could be home to c. 22% of the global population of the species (estimated in 2011 to be c. 197,000 individuals; [12]), although the global estimate did not include countries like the Gambia and Guinea-Bissau, where no information was available. Nevertheless, this reinforces the importance of this region, as previously reported for the Gambia and Guinea-Conakry [12,69,75]. Ultimately, this population estimate represent a broad, yet useful indication of theabundance of a globally threatened species in its main bastion of its whole distribution. This is especially relevant for the design of conservation plans, which often include numerical population objectives, and to inform the choice of priority sites for intervention.

### Exploring social aspects of vulture conservation

Our interviews suggest that 76% key stakeholders regard vultures as important providers of services and are aware of their benefits, especially for waste and carcass removal. This results are in line with those reported from interviews to butchers and meat buyers in Ghana [45] and to farmers in Namibia [25] but contrast with reports from Spain, where most farmers interviewed in a survey about ecosystem services provided by scavengers perceived scavengers has rather harmful [44]. These services are of great importance in a country like Guinea-Bissau, where waste management and carcass removal capacity are rudimentary, and there is a lot of organic waste left on the streets and healthcare services are poor. Thus, the proximity of vultures to humans may imply a reciprocal relationship that goes beyond the standard ‘commensalism’ proposed by Gangoso et al. [13] between Egyptian vultures and humans in Socotra, and may be more accurately described as ‘mutualism’(see also [44,76,77] for more examples on human-vulture relationship).

Most respondents regarded as negative the use of vultures as food source, unlike reported in other countries [34,78,79], since vultures were generally regarded as ‘non-edible’. This, associated with an overall positive perception of vultures by humans (c. 79% respondents), a generalised absence of antagonism towards these birds, and the apparent absence of the use of NSAID’s (such as diclofenac), might be some of the main reasons why vultures remain abundant in Guinea-Bissau.

Nonetheless, the physical proximity between vultures and people make them highly vulnerable to human-induced threats and the fact that >21% of the respondents expressed a negative opinion towards this species highlights the potential risks. Moreover, while persecution for sorcery and traditional medicine was most frequently recognised in the threat ranking exercise, many respondents (>28%) regarded this as a positive way of exploiting vultures or was not against it. As this practice has caused severe declines and even local extinctions in other West African countries [26,33,42,68,80], its mention during interviews should constitute a matter for concern. The evidence of a sudden collapse of Hooded vulture populations in Ghana in only 10 years, attributed mostly to transboundary trade for traditional medicine [68], is an example of the devastation that may come with this practice. Many respondents also referred to food shortage as a threat to vultures. Food shortage due to sanitary regulations in Europe have been linked to negative impacts on demographic parameters of vultures (delay in laying dates and regressive trends in clutch size, breeding success and survival, increase in mortality) [81], while there are several studies showing that rubbish dumps can modify movement patterns of several species which benefit from them, namely Hooded vultures [45,68,82–84]. In the light of these potential impacts, food shortage references should perhaps be further investigated.

## Conservation implications

The strong association between vultures and people provides an opportunity to monitor closely the state of populations, and offer a greater scope to control underlying threats. Intentional and indirect poisoning and persecution for body parts for sorcery remain the most important threats for vultures in West Africa [30,41,85], which we also report for Guinea-Bissau in this study. As such, these threats must be urgently tackled with a more exhaustive identification of areas of concern and focused awareness campaigns. Guinea-Bissau, like other West-African countries, is still a developing country, especially regarding basic sanitation and the industry. This provides a unique opportunity to include vulture conservation-related measures in the unavoidable upcoming evolution of sanitary regulations at abattoirs and dumpsites, and in carcass removal strategies, on which vultures seem to depend to such large extent [82,84,86,87]. Likewise, the apparent absence of veterinary NSAIDs like diclofenac, which seems to be the norm in all West African region [6,41], must be kept, as diclofenac is widely available in the market for treatment of human ailments and there is a risk of this human drug reaching the veterinary market [88]. The inclusion of a legal framework at a regional level (using regional binding organizations like the Economic Community of West African States–ECOWAS; e.g. see the European Union model and current problematics [89,90]) to ensure these drugs keep out of veterinary use is to be seriously considered. Other important measures to be considered are the inclusion of vulture-safe designs in future constructions of powerlines and poles and the conservation of big trees in construction planning, which are very important as roosts for Hooded vultures in urban areas, and as breeding and roosting sites for African white-backed and Rüppell’s vultures in rural areas. Finally, there is a need to monitor populations and assess trends, to better identify situations of potential conservation concern.

## Supporting information

S1 Appendix Methodological details of vulture population estimates. Methodological details of density estimates using strip transects in large cities, for vulture population estimates.
(DOCX)

S2 Appendix Stakeholder survey full questionnaire. Full questionnaire used in the stakeholder survey to assess the perception of stakeholders towards the cost and benefits of vultures and the perceived prevalence of human behaviours of concern to vulture conservation.
(DOCX)

S3 Appendix Veterinaries and livestock herders’ full questionnaire. Veterinaries and livestock herders’ full questionnaire to assess the potential presence and use of Non-Steroidal Anti-inflammatory Drugs (NSAID’s).
(DOCX)

S1 Table Description of the main characteristics of study participants. Table with the description of study participants interviewed to assess the social aspects of vulture conservation.
(DOCX)

S2 Table Results of roadside transects conducted through Guinea-Bissau. Raw data from roadside transects conducted throughout Guinea-Bissau.
(DOCX)

S3 Table Distribution of Hooded and *Gyps spp*. over habitat classes. Number of birds of *Hooded* and *Gyps ssp*. vultures sighted in association with one of nine habitat classes.
(DOCX)

S4 Table Results of transect counts in the cities of Bissau and Gabú. Raw data from bicycle transects conducted in the cities of Bissau and Gabú.
(DOCX)

S5 Table Results of human settlement counts of roosting Hooded vultures. Raw data from complete counts of roosting Hooded vultures in the human settlements sampled.
(DOCX)
