# Supplementary material for: Not in wilderness: African vulture strongholds remain in areas with high human density
Source: PLoS One. 2018 Jan 31;13(1):e0190594. doi: 10.1371/journal.pone.0190594 (PMC5791984; doi:10.1371/journal.pone.0190594)
Supplement: S1 Appendix — Methodological details of density estimates using strip transects in large cities, for vulture population estimates. (DOCX) [file pone.0190594.s001.docx]

**S1 Appendix.** **Methodological details of density estimates using strip transects in large cities, for vulture population estimates**

Data collected during bicycle transects in the city of Bissau and Gabú were used to estimate the density of Hooded vultures (birds per km^2^). We used the perpendicular distances recorded for each sighting to apply the strip transect methodology, in which we assume to spot every bird inside a given distance band. The sampled area is calculated multiplying the strip width by the total length of the transects and then, extrapolate the number of birds for all the surface area of each of the two cities [1–4]. To select which strip width to use for the estimation, we used data from Bissau to construct a graph with all the density estimates for strip widths of 10, 20, 30, 40, 50, 60, 70, 80, 90 and 100 meters (half of this value on each side of the transect; Fig. 1). Densities with very narrow strips likely underestimated true city densities, because comparatively few vultures use the road area, where the observer travelled. As the strip widens, the sides of the road and adjacent buildings and trees are included, where most landed/perched vultures are to be found, and hence density estimates increase. As the strip-with continues to broaden, more and more perched vultures are missed, hidden by the nearest buildings or trees, and hence estimated densities decline (Fig. 1).

The most stable area of the graph was selected and the strip width which produced the higher density estimate (60m, 30m each side of the observer) was chosen to extrapolate for the entire city. The same strip width was then used to also estimate the population of Hooded vultures in Gabú, based on transect counts conducted there.

Correction factor for Hooded vultures flying during low activity periods

Using the data from the activity pattern counts of Hooded vultures, published in Henriques et. al 2017 [5], we calculated a correction factor for birds that are flying during low activity periods (early morning and late afternoon). This correction factor is to be applied to the population estimates of Hooded vultures in the cities of Bissau and Gabú, since in the strip transect method we only account for perched birds. We calculated the mean number of Hooded vultures of all daily activity point counts between 7:20 and 8:20 (mean = 0.7 birds; SD = ± 2.9; n = 33) and between 17:30 and 19:20 (mean = 14.6 birds; SD = ± 16.5; n = 50), which corresponds to the mean of the number of birds flying during those periods. Then, we also calculated the mean of the birds flying during the most active period of the day, between 10:00 and 15:30 (mean = 71.6 birds; SD = ± 30.5; n = 102). The proportion of birds flying during the periods of the transects is given relatively to the mean of the number of birds flying during the most active period (proportion of early morning period = 1%; proportion of late afternoon period = 20.3%, mean = 10.65%). This results in a correction factor of 1.12, calculated from the following formula: Numb. vultures = Numb. vultures perched/(1-Mean proportion of vultures flying during low activity periods).

Calculations

The density of hooded vultures in the city of Bissau, for the chosen band width is approximately 77 birds per km^2^, equivalent to a total of 6,354 perched birds, and approximately 7,117 vultures, after applying the correction factor (1.12). Using the same band width for the calculations in city of Gabú, and based on the surveys conducted there, we estimate a density of approximately 102 birds per km^2^, which results in a total abundance estimate of 991 hooded vultures for that city.


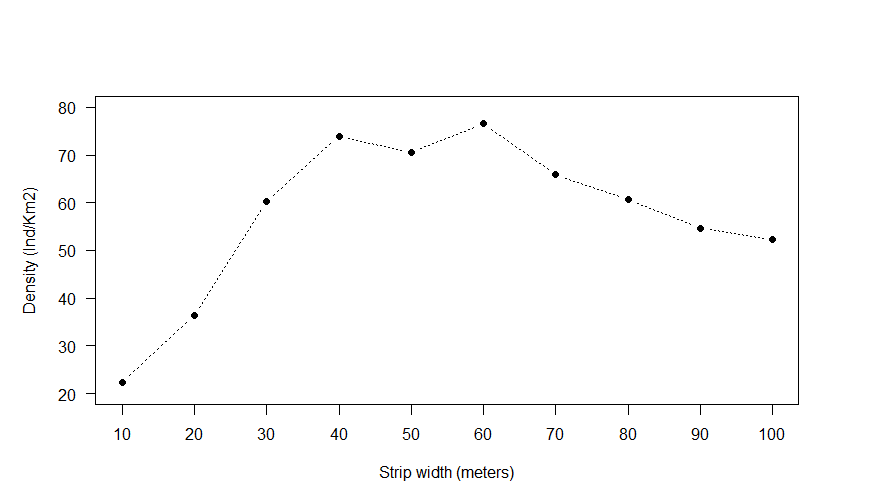


**Figure 1.** Representation of estimated densities of Hooded vultures in Bissau, based on several different strip widths and sampling areas. Density values for band widths of 40, 50 and 60 meters (3.4%, 4.3% and 5.2% of the area of the city sampled, respectively) remains relatively stable.

References

1. Carrascal LM, Seoane J, Palomino D. Bias in density estimations using strip transects in dry open-country environments in the Canary Islands. Anim Biodivers Conserv. 2008;31: 45–50.

2. García-del-Rey E. Density estimates of passerine bird species in Tenerifean coastal scrub using two different methods (Canary Islands). VIERAEA. 2005;33: 193–200.

3. Eberhardt LL. Transect methods for population studies. J Wildl Manage. 1978;42: 1–31.

4. Conner RN, Dickson JG. Strip transect sampling and analysis for avian habitat studies. Wildl Soc Bull. 1980;8: 4–10.

5. Henriques M, Lecoq M, Monteiro H, Granadeiro JP, Regalla A, Catry P. Status of birds of prey in Guinea-Bissau: first assessement based on road surveys. Ostrich. 2017;88: 101–111.
