# Supplementary material for: Not in wilderness: African vulture strongholds remain in areas with high human density
Source: PLoS One. 2018 Jan 31;13(1):e0190594. doi: 10.1371/journal.pone.0190594 (PMC5791984; doi:10.1371/journal.pone.0190594)
Supplement: S2 Appendix — Full questionnaire used in the stakeholder survey to assess the perception of stakeholders towards the cost and benefits of vultures and the perceived prevalence of human behaviours of concern to vulture conservation. (DOCX) [file pone.0190594.s002.docx]

**S2 Appendix. Stakeholder survey full questionnaire**

Hello, my name is (name) and I am collecting information for a study being conducted by the (institution).

We are conducting a study about how people perceive vultures in Guinea Bissau. We would like to ask you some questions. If you decide to answer these questions, this information will be held totally anonymously, so that it is impossible to trace this information back to you.

Participation in this study is entirely voluntary and you can withdraw from the study at any time without giving a reason. Any information provided will be shared with the research team and may be used in subsequent publications. A summary of the information will also be made public locally. This questionnaire will take approximately 25 minutes.

Would you like to participate in this study?

[If **NO**: please assess reason]

[If **YES**: continue with the questionnaire]

**1. What is your main occupation/s?**

**2. (Only for the village/town leaders) What is/are the main ethnic group/s in your village? There are other groups from other countries? If yes, which ones?**

**3. How important are vultures for you? You can mention negative and positive aspects.** (Probe for perspectives about socioeconomic, ecological and emotional values. Also, ask about importance for wider communities. If respondent says s/he doesn’t care about vultures, write that and probe for why that is the case)

**4. Cost/benefit exercise: Now, I’m going to mention a few reasons why people might think vultures are important in Guinea Bissau. Not all of these reasons are likely to apply to everyone so please let me know what you think.** (Fill in next table with comments)

| Type of service | For respondent | | For other people/groups | | |
| --- | --- | --- | --- | --- | --- |
|  | Positive effect? If so, how? | Negative effect? If so, how? | Positive effect? If so, how? | Negative effect? If so, how? | Who? |
| Food |  |  |  |  |  |
| Medicine |  |  |  |  |  |
| Cultural |  |  |  |  |  |
| Clean-up |  |  |  |  |  |
| Sorcery/ witchcraft |  |  | . |  |  |
| … |  |  |  |  |  |

1. **Do you know the Hooded vulture? And griffon vultures? If yes, in your opinion, over the last 10 years, Hooded and griffon vulture populations in Guinea Bissau have: increased, remained stable or decreased?** **Why?** (only consider answers from respondents that show to know the species in question)
2. **In your opinion, what are the main issues affecting vultures in Guinea Bissau?** (Probe for direct and indirect threats. If the respondent do not mention unintentional and indirect poisoning, and persecution for body parts for witchcraft, ask direct questions about these potential threats. Also ask about possible human-wildlife conflicts – such as hyenas or leopard attacking cattle – to get an indication of potential indirect poisoning)
3. **For each of the factors you listed, please complete table below. Definitions are as follows:**

**Spatial scope:** Defined as the proportion of the vulture's range likely to be negatively impacted by the direct and indirect threats

**Severity:** Defined as the level of impact of the direct and indirect threats

**Reversibility:** Defined as the capacity to recover from the effects of the direct and indirect threats

| **Type of threat** | **Spatial scope** | **Level of impact (severity)** | **Reversibility (capacity to recover)** |
| --- | --- | --- | --- |
| E.g. poaching |  |  |  |
|  |  |  |  |
|  |  |  |  |

**Note:** The criteria used for ranking spatial scope, severity and reversibility were as follows:

| **Value** | **Spatial scope** | **Level of impact (severity)** | **Reversibility (capacity to recover)** |
| --- | --- | --- | --- |
| 0 | Absent | No impact or minimal | Easily reversible |
| 1 | <25% | Moderately degrades | Reversible if enough commitment |
| 3 | 25-75% | Seriously degrades | Reversible but with great difficulty |
| 5 | >75% | Completely destroys or eliminates | Not reversible |
