# Supplementary material for: Not in wilderness: African vulture strongholds remain in areas with high human density
Source: PLoS One. 2018 Jan 31;13(1):e0190594. doi: 10.1371/journal.pone.0190594 (PMC5791984; doi:10.1371/journal.pone.0190594)
Supplement: S3 Appendix — Veterinaries and livestock herders’ full questionnaire to assess the potential presence and use of Non-Steroidal Anti-inflammatory Drugs (NSAID’s). (DOCX) [file pone.0190594.s003.docx]

**S3 Appendix. Veterinaries and livestock herders’ questionnaire table to assess the presence and use of Nonsteroidal Anti-inflammatory Drugs (NSAID’s) and other drugs that may be harmful to vultures**

Ask veterinaries and livestock herders that medicate their cattle about all the types of veterinary drugs they use. Fill in the table below for each drug mentioned by the respondent**.** Ask directly about diclofenac and NSAID’s in general when the respondent don’t mention it.

**Frequency of use is to be classified as follow:** 0-Never; 1- Once a year or less often; 2- A few times per year; 3- Monthly; 4- Weekly/ daily

| **Name of product** | **Function** | **Frequency of use** | **Known side-effects on livestock** | **Known effects on vultures** |
| --- | --- | --- | --- | --- |
|  |  |  |  |  |
|  |  |  |  |  |
|  |  |  |  |  |
