# Supplementary material for: Not in wilderness: African vulture strongholds remain in areas with high human density
Source: PLoS One. 2018 Jan 31;13(1):e0190594. doi: 10.1371/journal.pone.0190594 (PMC5791984; doi:10.1371/journal.pone.0190594)
Supplement: S1 Table — Table with the description of study participants interviewed to assess the social aspects of vulture conservation. (DOCX) [file pone.0190594.s004.docx]

**S1 Table.** Description of the main characteristics of study participants

**S1 Table.** Summary of the main characteristics of study participants (n=47 interviews to 74 respondents). Age does not include group interviews. ^a^ In each interview, respondents could name more than one occupation, thus total count exceeds sample size; ^b^ Group interview to three female veterinary inspectors at a slaughterhouse

| **Characteristics** | **Level** | **Count** |
| --- | --- | --- |
| Type of interview | Individual | 39 |
|  | Group | 8 |
| Occupation ^a^ | Local governmental representative | 2 |
|  | Butchers | 8 |
|  | Livestock-herders/Livestock-farmers | 8 |
|  | Farmers | 7 |
|  | Forester | 3 |
|  | Fishermen | 1 |
|  | Protected areas staff | 5 |
|  | Livestock Government Service | 2 |
|  | Slaughterhouse employees | 1 |
|  | Traditional leader | 7 |
|  | War veteran | 1 |
|  | Veterinaries | 8 |
|  | Witchcrafters/Spiritual leaders | 4 |
| Age | [20 – 35[ | 3 |
|  | [35 – 50[ | 10 |
|  | [50 – 65[ | 15 |
|  | > 65 | 8 |
|  | Unknown | 3 |
| Sex | M | 46 |
|  | F | 1^b^ |
| Sector | Bambadinca | 3 |
|  | Bigene | 1 |
|  | Quinhamel | 2 |
|  | Bissau | 6 |
|  | Boé | 3 |
|  | Bubaque | 5 |
|  | Cacheu | 2 |
|  | Cacine | 2 |
|  | Caió | 3 |
|  | Canchungo | 1 |
|  | Caravela | 4 |
|  | Gabú | 3 |
|  | Mansaba | 1 |
|  | Piche | 2 |
|  | Pirada | 3 |
|  | São-Domingos | 3 |
|  | Sonaco | 3 |
