# Supplementary material for: Not in wilderness: African vulture strongholds remain in areas with high human density
Source: PLoS One. 2018 Jan 31;13(1):e0190594. doi: 10.1371/journal.pone.0190594 (PMC5791984; doi:10.1371/journal.pone.0190594)
Supplement: S2 Table — Raw data from roadside transects conducted throughout Guinea-Bissau. (DOCX) [file pone.0190594.s005.docx]

**S2 Table: Results of roadside transects conducted through Guinea-Bissau.**

**S2 Table.** Data from roadside transects conducted throughout Guinea-Bissau, with encounter rates (measured in birds per km of transect) for each species/group of species in each Sector (an administrative division of the country).

| **Sector** | ***Necrosyrtes monachus*** | ***Gyps africanus*** | ***Gyps rueppelli*** | **Unidentified *Gyps sp.*** | **All *Gyps ssp.*** | **Transect size (km)** |
| --- | --- | --- | --- | --- | --- | --- |
| Bafata | 6.73 | 0.00 | 0.00 | 0.00 | 0.00 | 45.33 |
| Bambadinca | 5.42 | 0.00 | 0.00 | 0.00 | 0.00 | 63.65 |
| Bedanda | 0.30 | 0.00 | 0.00 | 0.00 | 0.00 | 40.34 |
| Bigene | 2.61 | 0.11 | 0.00 | 0.00 | 0.11 | 52.54 |
| Bissau | 27.19 | 0.14 | 0.00 | 0.00 | 0.14 | 13.90 |
| Bissora | 2.59 | 0.00 | 0.00 | 0.00 | 0.00 | 19.66 |
| Boe | 0.01 | 0.00 | 0.00 | 0.00 | 0.00 | 139.71 |
| Buba | 0.10 | 0.00 | 0.00 | 0.00 | 0.00 | 49.25 |
| Bula | 2.38 | 0.00 | 0.00 | 0.00 | 0.00 | 54.09 |
| Cacheu | 4.65 | 0.02 | 0.00 | 0.00 | 0.02 | 47.32 |
| Cacine | 1.04 | 0.00 | 0.00 | 0.00 | 0.00 | 37.38 |
| Caio | 6.00 | 0.00 | 0.00 | 0.00 | 0.00 | 12.00 |
| Canghungo | 10.56 | 0.33 | 0.03 | 0.00 | 0.37 | 65.69 |
| Catio | 2.74 | 0.04 | 0.00 | 0.00 | 0.04 | 25.90 |
| Empada | 0.37 | 0.00 | 0.00 | 0.00 | 0.00 | 40.14 |
| Fulacunda | 0.32 | 0.00 | 0.00 | 0.00 | 0.00 | 31.68 |
| Gabu | 5.56 | 0.02 | 0.00 | 0.01 | 0.03 | 93.40 |
| Galomaro | 1.56 | 0.17 | 0.00 | 0.07 | 0.24 | 96.13 |
| Gamamundo | 0.40 | 0.00 | 0.00 | 0.00 | 0.00 | 37.43 |
| Mansaba | 0.74 | 0.00 | 0.00 | 0.00 | 0.00 | 66.03 |
| Mansoa | 0.38 | 0.00 | 0.00 | 0.00 | 0.00 | 61.27 |
| Nhacra | 1.21 | 0.00 | 0.00 | 0.00 | 0.00 | 53.11 |
| Piche | 1.02 | 0.09 | 0.00 | 0.00 | 0.09 | 99.02 |
| Pirada | 1.07 | 0.01 | 0.00 | 0.28 | 0.30 | 70.18 |
| Prabis | 2.89 | 0.09 | 0.00 | 0.00 | 0.09 | 21.11 |
| Quebo | 0.60 | 0.00 | 0.00 | 0.00 | 0.00 | 112.33 |
| Quinhamel | 6.73 | 0.43 | 0.02 | 0.00 | 0.45 | 44.15 |
| Safim | 3.55 | 0.00 | 0.00 | 0.00 | 0.00 | 29.01 |
| Sao Domingos | 2.19 | 0.52 | 0.00 | 0.00 | 0.52 | 67.24 |
| Sonaco | 2.10 | 0.33 | 0.00 | 0.03 | 0.35 | 36.71 |
| Tite | 0.42 | 0.00 | 0.00 | 0.00 | 0.00 | 14.43 |
| Xitole | 1.04 | 0.00 | 0.00 | 0.00 | 0.00 | 71.20 |
| Bolama | NA | NA | NA | NA | NA | NA |
| Bubaque | NA | NA | NA | NA | NA | NA |
| Caravela | NA | NA | NA | NA | NA | NA |
| Contuboel | NA | NA | NA | NA | NA | NA |
| Farim | NA | NA | NA | NA | NA | NA |
