# Supplementary material for: Not in wilderness: African vulture strongholds remain in areas with high human density
Source: PLoS One. 2018 Jan 31;13(1):e0190594. doi: 10.1371/journal.pone.0190594 (PMC5791984; doi:10.1371/journal.pone.0190594)
Supplement: S3 Table — Number of birds of Hooded and Gyps ssp. vultures sighted in association with one of nine habitat classes. (DOCX) [file pone.0190594.s006.docx]

**S3 Table:** **Distribution of Hooded and Gyps spp. over habitat classes.**

**S3 Table.** Number of birds of *Hooded* and *Gyps ssp.* vultures sighted in association with one of nine habitat classes. NI stands for non-identified habitat, for sightings to which no habitat association could be confirmed.

| **Habitat** | ***Necrosyrtes monachus*** | **All *Gyps ssp.*** |
| --- | --- | --- |
| Bolanha | 148 | 1 |
| Cultivations | 5 | 0 |
| Forest | 112 | 4 |
| Human Settlements | 2744 | 48 |
| Lala | 33 | 5 |
| Mixed | 42 | 1 |
| NI | 765 | 29 |
| Orchards | 332 | 18 |
| Savannah | 106 | 49 |
| Wetlands | 73 | 4 |
