# Supplementary material for: Not in wilderness: African vulture strongholds remain in areas with high human density
Source: PLoS One. 2018 Jan 31;13(1):e0190594. doi: 10.1371/journal.pone.0190594 (PMC5791984; doi:10.1371/journal.pone.0190594)
Supplement: S4 Table — Raw data from bicycle transects conducted in the cities of Bissau and Gabú. (DOCX) [file pone.0190594.s007.docx]

**S4 Table: Results of transect counts in the cities of Bissau and Gabú.**

**S4 Table.** Raw data from bicycle transects conducted in the cities of Bissau and Gabú.

| Date | City | Transect ID | Transect size (m) | Start Time | End Time | Numb. ind | Distance |
| --- | --- | --- | --- | --- | --- | --- | --- |
| 12/02/2016 | Bissau | TRAN01 | 2114 | 07:20 | 07:40 | 3 | 96 |
|  |  |  |  |  |  | 7 | 20 |
|  |  |  |  |  |  | 2 | 40 |
|  |  |  |  |  |  | 4 | 44 |
|  |  |  |  |  |  | 2 | 80 |
|  |  |  |  |  |  | 1 | 80 |
|  |  |  |  |  |  | 9 | 84 |
|  |  |  |  |  |  | 3 | 90 |
|  |  |  |  |  |  | 9 | 160 |
|  |  | TRAN02 | 2264 | 07:45 | 08:00 | 1 | 10 |
|  |  |  |  |  |  | 2 | 30 |
|  |  |  |  |  |  | 2 | 30 |
|  |  |  |  |  |  | 1 | 33 |
|  |  | TRAN03 | 5150 | 18:08 | 18:56 | 1 | 16 |
|  |  |  |  |  |  | 1 | 145 |
|  |  |  |  |  |  | 3 | 0 |
|  |  |  |  |  |  | 2 | 0 |
|  |  |  |  |  |  | 5 | 5 |
|  |  |  |  |  |  | 1 | 5 |
|  |  |  |  |  |  | 4 | 7 |
|  |  |  |  |  |  | 3 | 15 |
|  |  |  |  |  |  | 1 | 20 |
|  |  |  |  |  |  | 2 | 25 |
|  |  |  |  |  |  | 13 | 25 |
|  |  |  |  |  |  | 3 | 25 |
|  |  |  |  |  |  | 1 | 30 |
|  |  |  |  |  |  | 1 | 30 |
|  |  |  |  |  |  | 6 | 30 |
|  |  |  |  |  |  | 1 | 30 |
|  |  |  |  |  |  | 4 | 30 |
|  |  |  |  |  |  | 16 | 60 |
|  |  |  |  |  |  | 3 | 75 |
|  |  |  |  |  |  | 2 | 100 |
|  |  |  |  |  |  | 29 | 125 |
|  |  |  |  |  |  | 31 | 125 |
|  |  |  |  |  |  | 2 | 150 |
|  |  |  |  |  |  | 1 | 155 |
|  |  |  |  |  |  | 1 | 220 |
| 13/02/2016 | Bissau | TRAN04 | 931 | 18:06 | 18:16 | 2 | 25 |
|  |  | TRAN05 | 1269 | 18:33 | 19:01 | 14 | 7 |
|  |  |  |  |  |  | 6 | 10 |
|  |  |  |  |  |  | 2 | 10 |
|  |  |  |  |  |  | 5 | 15 |
|  |  |  |  |  |  | 1 | 20 |
|  |  |  |  |  |  | 1 | 50 |
|  |  |  |  |  |  | 1 | 85 |
|  |  |  |  |  |  | 1 | 90 |
|  |  |  |  |  |  | 3 | 100 |
|  |  |  |  |  |  | 5 | 50 |
| 16/02/2016 | Bissau | TRAN06 | 2942 | 07:24 | 07:57 | 1 | 10 |
|  |  |  |  |  |  | 4 | 15 |
|  |  |  |  |  |  | 3 | 25 |
|  |  |  |  |  |  | 3 | 25 |
|  |  |  |  |  |  | 1 | 30 |
|  |  |  |  |  |  | 1 | 30 |
|  |  |  |  |  |  | 1 | 36 |
|  |  |  |  |  |  | 2 | 36 |
|  |  |  |  |  |  | 3 | 95 |
|  |  |  |  |  |  | 68 | 114 |
|  |  |  |  |  |  | 1 | 116 |
|  |  | TRAN07 | 3262 | 17:32 | 18:00 | 10 | 15 |
|  |  |  |  |  |  | 2 | 25 |
|  |  |  |  |  |  | 4 | 40 |
|  |  |  |  |  |  | 2 | 50 |
|  |  |  |  |  |  | 2 | 60 |
|  |  |  |  |  |  | 1 | 90 |
|  |  |  |  |  |  | 1 | 118 |
|  |  |  |  |  |  | 1 | 220 |
|  |  |  |  |  |  | 1 | 245 |
|  |  |  |  |  |  | 1 | 800 |
|  |  |  |  |  |  | 3 | 1500 |
|  |  | TRAN08 | 8409 | 18:00 | 18:57 | 1 | 28 |
|  |  |  |  |  |  | 4 | 50 |
|  |  |  |  |  |  | 3 | 75 |
|  |  |  |  |  |  | 3 | 75 |
|  |  |  |  |  |  | 6 | 185 |
|  |  |  |  |  |  | 1 | 185 |
|  |  |  |  |  |  | 3 | 200 |
|  |  |  |  |  |  | 1 | 210 |
|  |  |  |  |  |  | 2 | 230 |
|  |  |  |  |  |  | 1 | 300 |
| 17/02/2016 | Bissau | TRAN09 | 5142 | 17:56 | 19:00 | 1 | 0 |
|  |  |  |  |  |  | 1 | 20 |
|  |  |  |  |  |  | 67 | 20 |
|  |  |  |  |  |  | 2 | 37 |
|  |  |  |  |  |  | 8 | 50 |
|  |  |  |  |  |  | 1 | 76 |
|  |  |  |  |  |  | 3 | 125 |
|  |  |  |  |  |  | 1 | 130 |
|  |  |  |  |  |  | 1 | 260 |
|  |  |  |  |  |  | 1 | 800 |
|  |  |  |  |  |  | 3 | 1000 |
| 29/02/2016 | Bissau | TRAN10 | 6000 | 18:30 | 19:27 | 1 | 2 |
|  |  |  |  |  |  | 2 | 5 |
|  |  |  |  |  |  | 5 | 10 |
|  |  |  |  |  |  | 21 | 15 |
|  |  |  |  |  |  | 19 | 15 |
|  |  |  |  |  |  | 1 | 45 |
|  |  |  |  |  |  | 1 | 50 |
|  |  |  |  |  |  | 2 | 70 |
|  |  |  |  |  |  | 4 | 90 |
|  |  |  |  |  |  | 16 | 110 |
|  |  |  |  |  |  | 21 | 170 |
|  |  |  |  |  |  | 11 | 180 |
|  |  |  |  |  |  | 30 | 200 |
|  |  |  |  |  |  | 17 | 220 |
|  |  |  |  |  |  | 5 | 220 |
|  |  |  |  |  |  | 1 | 110 |
|  |  |  |  |  |  | 2 | 144 |
| 01/03/2016 | Bissau | TRAN11 | 6280 | 07:28 | 08:18 | 2 | 7 |
|  |  |  |  |  |  | 13 | 15 |
|  |  |  |  |  |  | 1 | 15 |
|  |  |  |  |  |  | 3 | 25 |
|  |  |  |  |  |  | 1 | 25 |
|  |  |  |  |  |  | 1 | 50 |
|  |  |  |  |  |  | 5 | 60 |
|  |  |  |  |  |  | 7 | 60 |
|  |  |  |  |  |  | 2 | 75 |
|  |  |  |  |  |  | 2 | 80 |
|  |  |  |  |  |  | 1 | 85 |
|  |  |  |  |  |  | 1 | 90 |
|  |  |  |  |  |  | 1 | 90 |
|  |  |  |  |  |  | 1 | 100 |
|  |  |  |  |  |  | 9 | 120 |
|  |  |  |  |  |  | 7 | 25 |
|  |  | TRAN12 | 11995 | 17:54 | 19:15 | 1 | 10 |
|  |  |  |  |  |  | 1 | 15 |
|  |  |  |  |  |  | 2 | 20 |
|  |  |  |  |  |  | 1 | 20 |
|  |  |  |  |  |  | 1 | 25 |
|  |  |  |  |  |  | 55 | 30 |
|  |  |  |  |  |  | 1 | 60 |
|  |  |  |  |  |  | 1 | 80 |
|  |  |  |  |  |  | 1 | 80 |
|  |  |  |  |  |  | 2 | 95 |
|  |  |  |  |  |  | 2 | 95 |
|  |  |  |  |  |  | 2 | 95 |
|  |  |  |  |  |  | 20 | 150 |
|  |  |  |  |  |  | 2 | 170 |
|  |  |  |  |  |  | 1 | 220 |
|  |  |  |  |  |  | 2 | 250 |
|  |  |  |  |  |  | 5 | 350 |
|  |  |  |  |  |  | 1 | 500 |
|  |  |  |  |  |  | 1 | 75 |
|  |  |  |  |  |  | 3 | 90 |
| 02/03/2016 | Bissau | TRAN13 | 6939 | 17:45 | 18:56 | 11 | 150 |
|  |  |  |  |  |  | 3 | 250 |
|  |  |  |  |  |  | 4 | 80 |
|  |  |  |  |  |  | 2 | 40 |
|  |  |  |  |  |  | 2 | 90 |
|  |  |  |  |  |  | 4 | 40 |
|  |  |  |  |  |  | 1 | 20 |
|  |  |  |  |  |  | 2 | 300 |
| 28/04/2016 | Bissau | TRAN15 | 8667 | 18:28 | 19:19 | 11 | 160 |
|  |  |  |  |  |  | 2 | 100 |
|  |  |  |  |  |  | 2 | 70 |
|  |  |  |  |  |  | 1 | 120 |
|  |  |  |  |  |  | 1 | 200 |
|  |  |  |  |  |  | 1 | 5 |
|  |  |  |  |  |  | 2 | 250 |
|  |  |  |  |  |  | 1 | 25 |
|  |  |  |  |  |  | 1 | 130 |
|  |  |  |  |  |  | 1 | 70 |
|  |  |  |  |  |  | 1 | 30 |
| 20/04/2016 | Gabu | TRANGAB01 | 5403 | 18:44 | 19:31 | 1 | 40 |
|  |  |  |  |  |  | 3 | 200 |
|  |  |  |  |  |  | 1 | 5 |
|  |  |  |  |  |  | 2 | 230 |
|  |  |  |  |  |  | 2 | 40 |
|  |  |  |  |  |  | 3 | 10 |
|  |  |  |  |  |  | 1 | 25 |
|  |  |  |  |  |  | 18 | 25 |
|  |  |  |  |  |  | 10 | 0 |
|  |  |  |  |  |  | 2 | 35 |
|  |  |  |  |  |  | 15 | 280 |
|  |  |  |  |  |  | 1 | 10 |
|  |  |  |  |  |  | 1 | 15 |
|  |  |  |  |  |  | 5 | 40 |
|  |  |  |  |  |  | 26 | 120 |
|  |  |  |  |  |  | 15 | 25 |
|  |  |  |  |  |  | 4 | 150 |
|  |  |  |  |  |  | 27 | 350 |
| 21/04/2016 | Gabu | TRANGAB02 | 9307 | 18:25 | 19:11 | 1 | 450 |
|  |  |  |  |  |  | 1 | 10 |
|  |  |  |  |  |  | 1 | 5 |
|  |  |  |  |  |  | 2 | 5 |
|  |  |  |  |  |  | 1 | 12 |
|  |  |  |  |  |  | 3 | 50 |
|  |  |  |  |  |  | 1 | 70 |
|  |  |  |  |  |  | 2 | 200 |
|  |  |  |  |  |  | 13 | 30 |
|  |  |  |  |  |  | 10 | 0 |
|  |  |  |  |  |  | 1 | 35 |
|  |  |  |  |  |  | 1 | 100 |
|  |  |  |  |  |  | 1 | 180 |
|  |  | TRANGAB03A | 2162 | 19:27 | 19:37 | 25 | 5 |
