# Supplementary material for: Not in wilderness: African vulture strongholds remain in areas with high human density
Source: PLoS One. 2018 Jan 31;13(1):e0190594. doi: 10.1371/journal.pone.0190594 (PMC5791984; doi:10.1371/journal.pone.0190594)
Supplement: S5 Table — Raw data from complete counts of roosting Hooded vultures in the human settlements sampled. (DOCX) [file pone.0190594.s008.docx]

**S5 Table: Results of human settlement counts of roosting Hooded vultures.**

**S5 Table.** Raw data from complete counts of roosting Hooded vultures in the human settlements sampled.

| **Human settlement** | **Number of *Necrosyrtes monachus*** | **Number of people** |
| --- | --- | --- |
| Cantchungo | 201 | 11600 |
| Bambadinca | 182 | 6437 |
| Bantandjã | 172 | 1773 |
| Caió | 101 | 1189 |
| Bubaque | 89 | 4299 |
| Sonaco | 60 | 2321 |
| Cacine | 58 | 977 |
| Safim | 51 | 2705 |
| Cacheu | 38 | 5674 |
| Pirada | 33 | 2512 |
| Jemberem | 28 | 992 |
| Buba | 26 | 7571 |
| Empada | 17 | 2267 |
| Mansaba | 14 | 5061 |
| Sao-Domingos | 80 | 5102 |
| Calequisse | 42 | 76 |
| Beli | 5 | 870 |
| Bijante Bubaque | 3 | 420 |
| Abu (Formosa) | 3 | 427 |
| Cumbidjã | 0 | 507 |
| Ilonde | 0 | 469 |
| Cabuca | 0 | 277 |
| Gã-Mamudo | 0 | 605 |
| Sintchan Sambel (Saltinho) | 36 | 958 |
| Madina Ali | 3 | 35 |
| Ponta Blimat | 0 | 555 |
| Ponta Nhara | 0 | 191 |
| Alto Fresco | 0 | 191 |
| Badique Felupe | 0 | 166 |
| Badique Manjaco | 0 | 85 |
| Bufa | 0 | 239 |
| Cassu | 1 | 192 |
